# Supplementary material for: Knowledge and competence in vestibular rehabilitation: a cross-sectional study of physical therapy interns
Source: PeerJ. 2025 Nov 4;13:e20213. doi: 10.7717/peerj.20213 (PMC12593722; doi:10.7717/peerj.20213)
Supplement: Supplemental Information 2 [file peerj-13-20213-s002.docx]

## **Data Collection Questionnair**

**A.** **Demographic and General Information:**

**Age:**

- 20-24
- 25-28
- 29-35

**Gender:**

- Male
- Female

**Professional Degree:**

- Doctor of Physical Therapy
- Physical Therapist

**Did you graduate from Saudi Arabia?**

- Yes (if yes please select below the list)
- No

**From which university did you graduate?**

- King Saud University
- Princess Nora bint Abdulrahman University
- AlMajmaah University
- Princes Sattam University
- Shaqra University
- Tabuk University
- Jouf University
- Hail University
- Taibah University
- Qassim University
- King Abdulaziz University
- Umm All Qura University
- Taif University
- Jazan University
- Najran University
- Imam Abdulrahman Bin Faisal University
- King Khalid University
- Burydah Private College
- Batterjee Medical College

**When did you start your Clinical Internship Rotation?**

- Less than a month
- Less than 3 months
- Less than 6 months
- More than 6 months
- More than 9 months

**Did you have Vestibular Rehabilitation courses during your undergraduate education?**

- Yes
- No

**From where did you learn Vestibular Rehabilitation? (You can choose more than one)**

- Formal university curriculum
- Workshops
- Self-learning
- Media (including social networking platforms)

1. **Awareness**

**Do you think that a physical therapist should be a member of a multidisciplinary team that manages patients with vestibular disorders?**

- Yes
- No

**Do you think that others health care professional are aware of physical therapy role in vestibular disorders?**

- Yes
- No

**Do you think that physical therapists can evaluate and treat patients with vestibular disorders?**

- Yes
- No

**Do you think that vestibular disorders have a significant impact on the well-being of individuals in society?**

- Yes
- No

1. **Training and clinical practice:**

**Have you seen patients with the vestibular disorders?**

- Yes
- No

**Have you seen patients complaining of dizziness?**

- yes
- No

**Do you feel confident that you can independently able to manage patients with vestibular disorder by vestibular rehabilitation?**

- Yes
- No

**Do you know how to perform a differential diagnosis and red flag identification in patients with vestibular disorders?**

- Yes
- No

**Identify the following tests and outcome measure that you learn during your academic years to assess patients with vestibular disorders? (You can choose more than one)**

- Dix-Hallpik test
- Roll test
- Side-lying test
- Head Impulse Test/ Head Thrust Test
- Head-Shaking Nystagmus Test
- Dynamic Visual Acuity Test
- Oculoomotor examination
- VOR Cancellation
- Modified Clinical Test of Sensory Interaction and Balance
- Dizziness Handicap Inventory scale
- Visual Vertigo Analog Scale
- Functional reach test
- Timed up and go test
- Dynamic gait index
- Functional gait assessment
- Star excursion balance test
- Berg balance scale
- Tinetti balance and mobility scale
- Dual-task
- ABC Scale (Tasks-specific balance confidence)
- None
- Others (Please specify) __________________________________________

**Identify the following tests and outcome measure that you learned during the internship and are used to assess patients with vestibular disorders?** **(You can choose more than one)**

- Dix-Hallpik test
- Roll test
- Side-lying test
- Head Impulse Test/ Head Thrust Test
- Head-Shaking Nystagmus Test
- Dynamic Visual Acuity Test
- Oculoomotor examination
- VOR Cancellation
- Modified Clinical Test of Sensory Interaction and Balance
- Dizziness Handicap Inventory scale
- Visual Vertigo Analog Scale
- Functional reach test
- Timed up and go test
- Dynamic gait index
- Functional gait assessment
- Star excursion balance test
- Berg balance scale
- Tinetti balance and mobility scale
- Dual-task tests
- ABC Scale (Tasks-specific balance confidence)
- None
- Others (Please specify) __________________________________________

**Identify the following maneuver that you learned during academic years to manage patients with vestibular disorders? (You can choose more than one)**

- Canalith Repositioning Maneuver / (Ebly) Maneuver
- Semont Maneuver
- Gufoni / Casani Maneuver
- Barbecue Roll Maneuver
- Imaginary Target Exercise
- Vestibulo-ocular reflex Adaptation 1 (VOR 1)
- Vestibulo-ocular reflex Adaptation 2 (VOR 2)
- Substitution Exercises: Active Eye Movements Between Two Targets
- Habituation Exercise (Brandt - Daroff Maneuver)
- Balance training
- Strength exercises
- Motor learning
- None
- Others (Please specify) __________________________________________

**Identify the following maneuvers that you learned during the internship and have used in patients with vestibular disorders? (You can choose more than one)**

- Canalith Repositioning Maneuver / (Ebly) Maneuver
- Semont Maneuver
- Gufoni / Casani Maneuver
- Barbecue Roll Maneuver
- Imaginary Target Exercise
- Vestibulo-ocular reflex Adaptation 1 (VOR 1)
- Vestibulo-ocular reflex Adaptation 2 (VOR 2)
- Substitution Exercises: Active Eye Movements Between Two Targets
- Habituation Exercise (Brandt - Daroff Maneuver)
- Balance training
- Strength exercises
- Motor learning
- None
- Others (Please specify) __________________________________________

**A 50 years old patient complaining of positional vertigo, he experiences when he is lying on the bed, and subsides after few seconds. His doctor examined him and cleared him medically?**

**Was the above more likely to be caused by:**

- Central lesion
- Peripheral lesion
- I am not sure

**A 60 years old female patient complaining of spinning sensation that she feels its attach while looking over her shoulder, CNS examination was unremarkable and patient was medically free?**

**Was the above more likely to be:**

- Vertical canal
- Horizontal canal
- I am not sure
